# Intense spectrally broad-band twin beams from poled nonlinear crystals

**DOI:** 10.1038/s41598-018-33546-0

**Published:** 2018-10-18

**Authors:** Jan Peřina

**Affiliations:** Joint Laboratory of Optics of Palacký University and Institute of Physics of the Czech Academy of Sciences, Faculty of Science, Palacký University, 17. listopadu 12, 77146 Olomouc, Czech Republic

## Abstract

Properties of intense twin beams generated in parametric down-conversion in periodically poled LiNbO_3_ crystals and their chirped variants by intense pump fields are analyzed along the model of intense parametric down-conversion that allows for pump depletion and uses the dual spatio-spectral Schmidt modes. Spectral and spatial intensity auto- and cross-correlation functions are determined as they depend on the pump power. Temporal correlations in intense twin beams at the fs time scale are investigated using the Hong-Ou-Mandel interferometer and the process of sum-frequency generation.

## Introduction

Periodical poling of nonlinear crystals with *χ*^(2)^ susceptibility, invented by Armstrong and his coworkers in 1962^1^, has become important and powerful tool in nonlinear optics in recent years^2^, mainly due to its application to LiNbO_3_^3,4^ and KTP crystals^5,6^. It allows for the compensation of natural nonlinear phase mismatch occurring among in general three nonlinearly interacting fields on one side, it enables to substantially modify the properties of these optical fields on the other side^2^. The ability to compensate for the natural phase mismatch opens the door for the exploitation of the largest nonlinear coefficients of a used material which considerably enhances the efficiency of nonlinear interaction under the required conditions. This enhancement has been used in second-harmonic generation^7–9^, difference-frequency generation^9,10^ and most frequently in parametric down-conversion for photon-pair generation^11–14^. The application of periodical poling to reach quasi-phase matching among three ordinary waves in the collinear geometry gives the so-called Type-0 three-mode interaction that is frequently used as a powerful source of entangled photon pairs, especially based on LiNbO_3_ crystals^13,15^. Global periodicity of the nonlinear grating introduced by poling that creates domains with differing signs of *χ*^(2)^ susceptibility can be replaced by local periodicity whose period may gradually change as we move along the crystal. This local change of the poling period known as chirped periodical (aperiodical) poling causes different phase matching conditions in different parts of a nonlinear crystal. Their suitable tailoring results in spectrally broad-band phase matching conditions^2^. Crystals poled this way then allow, e.g., for the generation of broad-band entangled photon pairs with spectral bandwidths extending over several hundreds of nm that are also endowed with ultra-fast temporal correlations at the fs time scale. Even photon pairs with correlation (entanglement) times comparable to one optical cycle can be produced^12,16,17^. These ultra-fast correlations are useful in metrology (measurement of ultra-short time delays^18^) and sample characterization (optical quantum coherence tomography^19^). For reaching ultra-short temporal correlations, careful compensation of relative spectral phases between the signal and idler fields is necessary; squared hyperbolic poling profiles perform the best here^20,21^. On the other hand, their broad-band spectra are promising for parallel quantum information processing^22,23^. Also other characteristic patterns of nonlinear domains have been found useful for specific purposes, e.g. for reaching higher-order quasi-phase matching^24^, generating spectrally broad-band squeezed light^20^ or simultaneous reaching of two kinds of phase matching conditions in more complex nonlinear interactions^25^. Also stochastically poled nonlinear structures^9,25–27^ and randomly poled nonlinear structures^15,28^ have been analyzed as sources of entangled photon pairs.

As the poled nonlinear crystals allow for efficient nonlinear interactions they are naturally suitable for the generation of more intense fields arising in the interaction. From the physical point of view, the generation of more intense fields endowed with quantum properties is the most appealing. Intense twin beams exhibiting sub-shot-noise intensity correlations and commonly generated in standard nonlinear crystals by intensively-pumped parametric down-conversion^29–34^ represent a typical example of such fields that could benefit from the properties of poled crystals. Indeed, usefulness of poled crystals for the generation of broad-band twin beams has already been experimentally demonstrated in^21^. Here, we continue this investigation by theoretically analyzing both spatial and spectral properties of intense twin beams in the regime with pump depletion. For this analysis, we generalize the original model of refs^35–38^ to include also poled nonlinear crystals. The obtained model is applied, as an example, to two typical poled LiNbO_3_ crystals, one having a regular grating, the other being chirped. In the model both coherence of the twin beam as well as its mode structure are determined. The model reveals coherence maxima^33,34^ observed both in spectral and spatial intensity auto- and cross-correlation functions for specific pump powers. They occur as a consequence of the reduction of the number of twin-beam modes arising in the nonlinear interaction. The model also predicts ultra-short temporal correlations in twin beams whose duration can be comparable to the time interval of one optical cycle in case of chirped crystals.

These intense twin beams may find their application in metrology as they are more resistant against the noise compared to their low intensity counterparts containing just one photon pair. They have been recently discussed as promising sources for the so-called virtual-state spectroscopy^39^ where they allow to increase the absolute two-photon absorption probabilities, that lie in the heart of the method, by four orders in magnitude^40^.

The paper is organized as follows. In Sec. II, the model of intense parametric down-conversion is described. Also quantities characterizing the generated twin beams are introduced. Mode structure and intensity parameters of the generated twin beams are discussed in Sec. III for two typical poled structures. Spectral and spatial properties of the twin beams are analyzed in Sec. IV. Temporal correlations in the twin beams and their behavior in the Hong-Ou-Mandel interferometer and the process of sum-frequency generation are in the center of attention in Sec. V. Conclusions are drawn in Sec. VI.

## Evolution of Intense Twin Beams

Intense twin beams are assumed to be generated in optical parametric down-conversion occurring in a poled nonlinear crystal with the spatially varying tensor *χ*^(2)^(*z*) of second-order nonlinear susceptibility. The appropriate interaction momentum operator ${\hat{G}}_{{\rm{int}}}$ for the signal (index s), idler (i) and pump (p) fields propagating along the *z* axis is expressed as follows^41,42^:

1 $${\hat{G}}_{{\rm{int}}}(z)=2{\varepsilon }_{0}\,\int \,dxdy\,{\int }_{-\infty }^{\infty }\,dt\,[{\chi }^{(2)}(z):{E}_{{\rm{p}}}^{(+)}({\bf{r}},t){\hat{E}}_{{\rm{s}}}^{(-)}({\bf{r}},t){\hat{E}}_{{\rm{i}}}^{(-)}({\bf{r}},t)+{\rm{H}}.\,{\rm{c}}.];$$

**r** = (*x*, *y*, *z*). The positive-frequency part of a classical pump electric-field amplitude is denoted as ${E}_{{\rm{p}}}^{(+)}$ whereas symbol ${\hat{E}}_{{\rm{s}}}^{(-)}$ [${\hat{E}}_{{\rm{i}}}^{(-)}$] stands for the negative-frequency part of a signal [idler] electric-field operator amplitude. Symbol *ε*_0_ stands for permittivity of vacuum, H.c. replaces the Hermitian conjugated term and symbol: shorthands the tensor with respect to its three indices. The poled nonlinear crystal is assumed to be composed of domains with negative susceptibility *χ*^(2)^ that are sandwiched by domains with positive susceptibility *χ*^(2)^. The boundaries between the domains occur at positions *z*_*j*_ given as

2 $${z}_{j}={L}_{0}+j{l}_{0}+\zeta ^{\prime} {(j-{N}_{L}/2)}^{2}{l}_{0}^{2}$$

where *ζ*′ = *ζ*/Δ*k*_0_, Δ*k*_0_ stands for the natural nonlinear phase mismatch at the central frequencies (${\rm{\Delta }}{k}_{0}={k}_{{\rm{p}}}^{0}-{k}_{{\rm{s}}}^{0}-{k}_{{\rm{i}}}^{0}$), *ζ* denotes the chirping parameter and *N*_*L*_ gives the number of domains. Parameter ${l}_{0}\equiv \pi /{\rm{\Delta }}{k}_{0}$ guarantees quasi-phase-matching and it means an average domain length. The crystal is positioned such that its end occurs at ${z}_{{N}_{L}}=0$ and so the constant *L*_0_ introduced in Eq. (2) equals $-{N}_{L}{l}_{0}-\zeta ^{\prime} {N}_{L}^{2}{l}_{0}^{2}/4$. The crystal length *L* is then given as *N*_*L*_*l*_0_. Periodically poled crystals are characterized by *ζ* = 0.

To describe the interacting fields through their temporal and spatial spectra, we express their electric-field amplitudes ${E}_{a}^{(+)}({\bf{r}},t)$ [${E}_{a}^{(+)}({\bf{r}},t)={E}_{a}^{(-)\ast }({\bf{r}},t)$] decomposed into harmonic plane waves with wave vectors **k**_*a*_(*ω*_*a*_) and frequencies *ω*_*a*_:

3 $${E}_{a}^{(+)}({\bf{r}},t)=\tfrac{1}{{\sqrt{2\pi }}^{3}c}\,\int \,d{{\bf{k}}}_{a}^{\perp }\,\int \,d{\omega }_{a}\,{E}_{a}^{(+)}({{\bf{k}}}_{a}^{\perp },{\omega }_{a})\,\exp (i{{\bf{k}}}_{a}({\omega }_{a}){\bf{r}}-i{\omega }_{a}t),\,a=p,s,i.$$

In Eq. (3), *c* is the speed of light in vacuum, ${{\bf{k}}}_{a}^{\perp }$ denotes the transverse part of wave vector **k**_*a*_ and the paraxial approximation is invoked when describing the fields’ propagation.

An incident pump field with amplitude *ξ*_p_ and central frequency ${\omega }_{{\rm{p}}}^{0}$ is assumed with Gaussian transverse profile (beam radius *w*_p_) and Gaussian temporal spectrum (pulse duration *τ*_p_):

4 $${E}_{{\rm{p}}}^{(+)}({{\bf{k}}}_{{\rm{p}}}^{\perp },{\omega }_{{\rm{p}}})={\xi }_{{\rm{p}}}\tfrac{\sqrt{{\tau }_{{\rm{p}}}}{w}_{{\rm{p}}}}{{(2\pi )}^{3/4}}\,\exp \,[-\tfrac{{w}_{{\rm{p}}}^{2}({k}_{{\rm{p}},{\rm{x}}}^{2}+{k}_{{\rm{p}},{\rm{y}}}^{2})}{4}]\,\exp \,[-\tfrac{{\tau }_{{\rm{p}}}^{2}{({\omega }_{{\rm{p}}}-{\omega }_{{\rm{p}}}^{0})}^{2}}{4}].$$

For a pulsed pump field with power *P* and repetition rate *f*, the incident amplitude *ξ*_p_ is determined as $\sqrt{P{\omega }_{{\rm{p}}}}/\sqrt{{\varepsilon }_{0}{c}^{2}{k}_{{\rm{p}}}\,f}$.

On the other hand, the spectral electric-field operator amplitudes ${E}_{a}^{(+)}({{\bf{k}}}_{a}^{\perp },{\omega }_{a})$, *a* = s, i, of the quantized signal and idler fields can conveniently be expressed in the dual bases of the Schmidt spectral [*f*_*a*,*q*_(*ω*_*a*_)] and spatial [${t}_{a,ml}({{\bf{k}}}_{a}^{\perp },{\phi }_{a})$] modes (for details, see^36^):

5 $${E}_{a}^{(+)}({k}_{a}^{\perp },{\phi }_{a},{\omega }_{a})=i\sqrt{\frac{\hslash {\omega }_{a}^{2}}{2{\varepsilon }_{0}{c}^{2}{k}_{a}}}\,\sum _{m=-\infty }^{\infty }\,\sum _{l,q=0}^{\infty }\,{t}_{a,ml}({k}_{a}^{\perp },{\phi }_{a}){f}_{a,q}({\omega }_{a}){\hat{a}}_{a,mlq}.$$

In Eq. (5), the introduced annihilation operators ${\hat{a}}_{a,mlq}$ are associated with the spatio-spectral modes ${t}_{a,ml}({k}_{a}^{\perp },{\phi }_{a}){f}_{a,q}({\omega }_{a})$ and *ħ* means the reduced Planck constant.

In the generalized parametric approximation that considers a classical pump field that depletes during the propagation the interaction momentum operator ${\hat{G}}_{{\rm{int}}}$ in Eq. (1) can approximately be replaced by the following momentum operator ${\hat{G}}_{{\rm{int}}}^{{\rm{av}}}$ written in the Schmidt-mode operators ${\hat{a}}_{a,mlq}$^36^:

6 $${\hat{G}}_{{\rm{int}}}^{{\rm{av}}}(z)=i\hslash \,\sum _{m=-\infty }^{\infty }\,\sum _{l,q=0}^{\infty }\,{K}_{mlq}^{{\rm{p}}}(z){\hat{a}}_{{\rm{s}},mlq}^{\dagger }(z){\hat{a}}_{{\rm{i}},mlq}^{\dagger }(z)+{\rm{H}}{\rm{.c}}{\rm{.}}$$

The coupling constants ${K}_{mlq}^{{\rm{p}}}(z)$ are linearly proportional to *χ*^(2)^ susceptibility and *z*-dependent pump-field amplitude *A*_p,*mlq*_(*z*)^36,37^. They can be written as ${K}_{mlq}^{{\rm{p}}}(z)\equiv {K}_{q}\,{A}_{{\rm{p}},mlq}(z)$, where ${K}_{q}\equiv ({\kappa }_{q}^{\parallel }{t}^{\perp }{f}^{\parallel }/L)\,({\xi }_{{\rm{p}}}/{\xi }_{{\rm{p}}}^{({\rm{n}})})$, ${({\kappa }_{q}^{\parallel })}^{2}$ gives the norm of spectral pump-field mode associated with a *q*-th signal-idler Schmidt dual mode, ${t}^{\perp }$ (${f}^{\parallel }$) quantifies the common nonlinear coupling constants among spatial (spectral) modes, *L* stands for the crystal length and ${\xi }_{{\rm{p}}}^{({\rm{n}})}=\sqrt{P/(f\hslash {\omega }_{{\rm{p}}}^{0})}$ is the overall pump-field amplitude expressed in photon numbers. We note that the spectral pump-field mode profile associated with a given dual signal- and idler-field Schmidt mode is determined by spectral convolution and its proper normalization is guaranteed by constants ${\kappa }_{q}^{\parallel }$^37^. The obtained pump-field modes are slightly non-orthogonal. The neighbor modes exhibit little overlap that, however, tends fast to zero with the increasing mode distance.

The generalized parametric approximation provides the pump-field amplitude *A*_p,*mlq*_ along the *z* axis in the form:

7 $${A}_{{\rm{p}},mlq}(z^{\prime} )={A}_{mlq}^{{\rm{ps}}}\frac{{A}_{mlq}^{{\rm{p}}}\,\cosh ({K}_{q}\,{A}_{mlq}^{{\rm{ps}}}z^{\prime} )-{A}_{mlq}^{{\rm{ps}}}\,\sinh ({K}_{q}\,{A}_{mlq}^{{\rm{ps}}}z^{\prime} )}{{A}_{mlq}^{{\rm{ps}}}\,\cosh ({K}_{q}\,{A}_{mlq}^{{\rm{ps}}}z^{\prime} )-{A}_{mlq}^{{\rm{p}}}\,\sinh ({K}_{q}\,{A}_{mlq}^{{\rm{ps}}}z^{\prime} )},$$

$z^{\prime} \equiv z+L$, ${A}_{mlq}^{{\rm{p}}}\equiv \sqrt{{({A}_{mlq}^{{\rm{p}}{\mathscr{N}}})}^{2}+1/2}$, ${A}_{mlq}^{{\rm{ps}}}\equiv \sqrt{{({A}_{mlq}^{{\rm{p}}{\mathscr{N}}})}^{2}+1}$ and ${A}_{mlq}^{{\rm{p}}{\mathscr{N}}}\equiv {\kappa }^{\parallel }({\lambda }_{ml}^{\perp }{\lambda }_{q}^{\parallel }/{\kappa }_{q}^{\parallel }){\xi }_{{\rm{p}}}^{({\rm{n}})}$ gives the incident pump-field amplitude of mode *mlq* related to normal ordering of field operators. Symbol ${\lambda }_{q}^{\parallel }$ (${\lambda }_{ml}^{\perp }$) stands for the spectral (spatial) Schmidt eigenvalue of mode *q* (*ml*) and ${\kappa }^{\parallel }=1/\sqrt{{\sum }_{q}\,{({\lambda }_{q}^{\parallel }/{\kappa }_{q}^{\parallel })}^{2}}$ is an appropriate normalization constant. We note that formula (7) is valid for positions *z*′ smaller than $z{^{\prime} }_{0,mlq}$ at which reversion of the nonlinear dynamics occurs^37^,

8 $$z{^{\prime} }_{0,mlq}=\frac{1}{2{K}_{q}\,{A}_{mlq}^{{\rm{ps}}}}\,\mathrm{ln}\,[1+\frac{2{A}_{mlq}^{{\rm{ps}}}}{{A}_{mlq}^{{\rm{ps}}}+1/\sqrt{2}}\frac{{A}_{mlq}^{{\rm{p}}}-1/\sqrt{2}}{{A}_{mlq}^{{\rm{ps}}}-{A}_{mlq}^{{\rm{p}}}}].$$

The momentum operator ${\hat{G}}_{{\rm{int}}}^{{\rm{av}}}(z)$ from Eq. (6) gives the following linear Heisenberg equations for each mode *mlq*:

9 $$\frac{d{\hat{a}}_{{\rm{s}},mlq}(z^{\prime} )}{dz^{\prime} }={K}_{mlq}^{{\rm{p}}}(z^{\prime} ){\hat{a}}_{{\rm{i}},mlq}^{\dagger }(z^{\prime} ),\,\frac{d{\hat{a}}_{{\rm{i}},mlq}(z^{\prime} )}{dz^{\prime} }={K}_{mlq}^{{\rm{p}}}(z^{\prime} ){\hat{a}}_{{\rm{s}},mlq}^{\dagger }(z^{\prime} ).$$

Their solution can be written in the following simple form:

10 $$\begin{array}{rcl}{\hat{a}}_{{\rm{s}},mlq}(z^{\prime} ) & = & {U}_{mlq}(z^{\prime} ){\hat{a}}_{{\rm{s}},mlq}(z^{\prime} =0)+{V}_{mlq}(z^{\prime} ){\hat{a}}_{{\rm{i}},mlq}^{\dagger }(z^{\prime} =0),\\ {\hat{a}}_{{\rm{i}},mlq}(z^{\prime} ) & = & {U}_{mlq}(z^{\prime} ){\hat{a}}_{{\rm{i}},mlq}(z^{\prime} =0)+{V}_{mlq}(z^{\prime} ){\hat{a}}_{{\rm{s}},mlq}^{\dagger }(z^{\prime} =0)\end{array}$$

and

11 $${U}_{mlq}(z^{\prime} )=\,\cosh \,[{\varphi }_{mlq}(z^{\prime} )],\,{V}_{mlq}(z^{\prime} )=\,\sinh \,[{\varphi }_{mlq}(z^{\prime} )],$$

12 $${\varphi }_{mlq}(z^{\prime} )={K}_{q}\,{A}_{mlq}^{{\rm{ps}}}z^{\prime} -\,\mathrm{ln}\,[\frac{{A}_{mlq}^{{\rm{ps}}}+{A}_{mlq}^{{\rm{p}}}}{2{A}_{mlq}^{{\rm{ps}}}}+\frac{{A}_{mlq}^{{\rm{ps}}}-{A}_{mlq}^{{\rm{p}}}}{2{A}_{mlq}^{{\rm{ps}}}}\,\exp (2{K}_{q}\,{A}_{mlq}^{{\rm{ps}}}z)].$$

The solution in Eq. (11) predicts thermal statistics of the emitted signal and idler fields originating in the incident vacuum state. We note that the occurrence of coherent component in the otherwise thermal statistics is anticipated when more general three-mode nonlinear momentum operators are considered^38^.

The emitted twin beam is characterized by several simple quantities. Individual fields are described by their spatio-temporal spectral photon-number densities *n*_*a*_ determined along the following formula

13 $${n}_{a}({k}_{a}^{\perp },{\phi }_{a},{\omega }_{a})=\langle {\hat{n}}_{a}({k}_{a}^{\perp },{\phi }_{a},{\omega }_{a})\rangle ,\,a=s,i,$$

that uses the photon-number operator ${\hat{n}}_{a}\equiv {\hat{a}}_{a}^{\dagger }(z^{\prime} =L){\hat{a}}_{a}(z^{\prime} =L)$ defined at the crystal end. The operators ${\hat{a}}_{a}$, *a* = s, i, are given in general as:

14 $${\hat{a}}_{a}({k}_{a}^{\perp },{\phi }_{a},{\omega }_{a},z^{\prime} )=\sum _{mlq}\,{t}_{a,ml}({k}_{a}^{\perp },{\phi }_{a}){f}_{a,q}({\omega }_{a}){\hat{a}}_{a,mlq}(z^{\prime} )$$

and symbol $\langle \rangle$ stands for quantum mechanical averaging. Substituting the solution in Eq. (10) into Eq. (13), we get the photon-number density *n*_*a*_ in field *a* in the following form:

15 $${n}_{a}({k}_{a}^{\perp },{\phi }_{a},{\omega }_{a})=\sum _{mlq}\,|{t}_{a,ml}({k}_{a}^{\perp },{\phi }_{a}){f}_{a,q}({\omega }_{a}){|}^{2}{V}_{mlq}^{2}$$

and coefficients *V*_*mlq*_ are taken at *z* = 0. Integration of the photon-number density *n*_*a*_ in Eq. (15) over the variables ${k}_{a}^{\perp }$ and *φ*_*a*_ in the transverse plane provides the temporal spectral intensity *I*_*a*,*ω*_ in the form:

16 $${I}_{a,\omega }({\omega }_{a})=\sum _{mlq}\,|\,{f}_{a,q}({\omega }_{a}){|}^{2}{V}_{mlq}^{2}.$$

Similarly, the formula for spatial spectral intensity *I*_*a*,*k*_ is obtained in the form:

17 $${I}_{a,k}({k}_{a}^{\perp },{\phi }_{a})=\sum _{mlq}\,|{t}_{a,ml}({k}_{a}^{\perp },{\phi }_{a}){|}^{2}{V}_{mlq}^{2}.$$

Also, the formula for overall intensity *I*_*a*_ of field *a*, *a* = s, i, expressed in the number of emitted photons is derived as:

18 $${I}_{a}=\sum _{mlq}\,{V}_{mlq}^{2}.$$

Intensity auto- (*A*) and cross- (*C*) correlation functions are the most important quantities describing coherence of intense twin beams. They are defined as follows using the normally-ordered (${\mathscr{N}}::$) intensity fluctuations ${\rm{\Delta }}\hat{n}$:

19 $$\begin{array}{ccc}{A}_{a}({k}_{a}^{\perp },{\phi }_{a},{\omega }_{a},{k}_{a}^{{}^{{\rm{^{\prime} }}}\perp },{\phi }_{a}^{{}^{{\rm{^{\prime} }}}},{\omega }_{a}^{{}^{{\rm{^{\prime} }}}}) & \equiv  & \langle {\mathscr{N}}\,:{\rm{\Delta }}{\hat{n}}_{a}({k}_{a}^{\perp },{\phi }_{a},{\omega }_{a}){\rm{\Delta }}{\hat{n}}_{a}({k}_{a}^{{}^{{\rm{^{\prime} }}}\perp },{\phi }_{a}^{{}^{{\rm{^{\prime} }}}},{\omega }_{a}^{{}^{{\rm{^{\prime} }}}}):\,\rangle ,\\ C({k}_{{\rm{s}}}^{\perp },{\phi }_{{\rm{s}}},{\omega }_{{\rm{s}}},{k}_{{\rm{i}}}^{\perp },{\phi }_{{\rm{i}}},{\omega }_{{\rm{i}}}) & \equiv  & \langle {\mathscr{N}}\,:{\rm{\Delta }}{\hat{n}}_{{\rm{s}}}({k}_{{\rm{s}}}^{\perp },{\phi }_{{\rm{s}}},{\omega }_{{\rm{s}}}){\rm{\Delta }}{\hat{n}}_{{\rm{i}}}({k}_{{\rm{i}}}^{\perp },{\phi }_{{\rm{i}}},{\omega }_{{\rm{i}}}):\,\rangle .\end{array}$$

Substituting Eq. (14) into Eq. (19), we arrive at the explicit formulas for the intensity auto- and cross-correlation functions:

20 $$\begin{array}{rcl}{A}_{a}({k}_{a}^{\perp },{\phi }_{a},{\omega }_{a},{k}_{a}^{^{\prime} \perp },{\phi }_{a}^{^{\prime} },{\omega }_{a}^{^{\prime} }) & = & {|\sum _{mlq}{t}_{a,ml}^{\ast }({k}_{a}^{\perp },{\phi }_{a}){f}_{a,q}^{\ast }({\omega }_{a}){t}_{a,ml}({k}_{a}^{^{\prime} \perp },{\phi }_{a}^{^{\prime} }){f}_{a,q}({\omega }_{a}^{^{\prime} }){V}_{mlq}^{2}|}^{2},\\ C({k}_{{\rm{s}}}^{\perp },{\phi }_{{\rm{s}}},{\omega }_{{\rm{s}}},{k}_{{\rm{i}}}^{\perp },{\phi }_{{\rm{i}}},{\omega }_{{\rm{i}}}) & = & {|\sum _{mlq}{t}_{{\rm{s}},ml}({k}_{{\rm{s}}}^{\perp },{\phi }_{{\rm{s}}}){f}_{{\rm{s}},q}({\omega }_{{\rm{s}}}){t}_{{\rm{i}},ml}({k}_{{\rm{i}}}^{\perp },{\phi }_{{\rm{i}}}){f}_{{\rm{i}},q}({\omega }_{{\rm{i}}}){U}_{mlq}{V}_{mlq}|}^{2}.\end{array}$$

Specific intensity auto- and cross-correlation functions characterizing the twin beam either in the spatial or temporal spectral domain are derived using appropriate averaging in Eq. (20).

Temporal counterparts $\tilde{f}$ of the spectral Schmidt functions *f* defined as

21 $${\tilde{f}}_{a,q}({t}_{a})=\sqrt{\frac{\hslash }{2\pi }}\,\int \,d{\omega }_{a}\sqrt{{\omega }_{a}}{f}_{a,q}({\omega }_{a})\,\exp (\,-\,i{\omega }_{a}{t}_{a})$$

allow us to describe temporal properties of the analyzed twin beam including its photon fluxes and photon-flux auto- and cross-correlation functions along the same vein as in the spectrum^36^. As temporal correlations in a twin beam are usually too fast to be directly measurable, other experimental approaches are used instead. Also in these cases the temporal functions $\tilde{f}$ allow for appropriate description of the observed quantities.

Detection of intensity arising in the process of sum-frequency generation^43^ seeded by the signal and idler fields^44^ represents the most common approach. Temporal correlations in the twin beam are deduced from the sum-frequency intensity profile *I *^SFG^ depending on the mutual delay *τ* between both fields and it is derived in the form:

22 $${I}^{{\rm{SFG}}}(\tau )=\eta \,{\int }_{-\infty }^{\infty }\,dt{\langle {\hat{E}}_{{\rm{s}}}^{(-)}(t+\tau ){\hat{E}}_{{\rm{i}}}^{(-)}(t){\hat{E}}_{{\rm{s}}}^{(+)}(t+\tau ){\hat{E}}_{{\rm{i}}}^{(+)}(t)\rangle }_{\perp }.$$

In Eq. (22), *η* is a suitable constant and subscript $\perp$ indicates averaging of the sum-frequency intensity over the transverse plane.

Alternatively, the Hong-Ou-Mandel interferometer^45^ and detection of correlations of intensity fluctuations at detectors *A* and *B* of the interferometer can be used to infer the information about temporal correlations in the twin beam. In this case, the interference pattern *R*^Δ^ that quantifies these correlations and depends on the mutual delay *τ* between the signal and idler fields is expressed as

23 $${R}^{{\rm{\Delta }}}(\tau )=\frac{4{\varepsilon }_{0}^{2}{c}^{2}}{{\hslash }^{2}{\omega }_{{\rm{s}}}^{0}{\omega }_{{\rm{i}}}^{0}}\,{\int }_{-{\rm{\infty }}}^{{\rm{\infty }}}\,d{t}_{{\rm{A}}}\,{\int }_{-{\rm{\infty }}}^{{\rm{\infty }}}\,d{t}_{{\rm{B}}}{\langle {\mathscr{N}}:{\rm{\Delta }}[{\hat{E}}_{{\rm{A}}}^{(-)}({t}_{{\rm{A}}}){\hat{E}}_{{\rm{A}}}^{(+)}({t}_{{\rm{A}}})]{\rm{\Delta }}[{\hat{E}}_{{\rm{B}}}^{(-)}({t}_{{\rm{B}}}){\hat{E}}_{{\rm{B}}}^{(+)}({t}_{{\rm{B}}})]:\rangle }_{\perp }.$$

The detector operator amplitudes ${\hat{E}}_{{\rm{A}}}^{(+)}$ and ${\hat{E}}_{{\rm{B}}}^{(+)}$ are derived from the mutually delayed signal and idler operator amplitudes ${\hat{E}}_{{\rm{s}}}^{(+)}$ and ${\hat{E}}_{{\rm{i}}}^{(+)}$ using transmissivity *t* and reflectivity *r* of the beam splitter that is placed in the interferometer:

24 $${\hat{E}}_{{\rm{A}}}^{(+)}(t)=r{\hat{E}}_{{\rm{i}}}^{(+)}(t)+t{\hat{E}}_{{\rm{s}}}^{(+)}(t-\tau ),\,{\hat{E}}_{{\rm{B}}}^{(+)}(t)={t}^{\ast }{\hat{E}}_{{\rm{i}}}^{(+)}(t)-{r}^{\ast }{\hat{E}}_{{\rm{s}}}^{(+)}(t-\tau ).$$

Finally, the twin beam changes the number *K* of effectively populated modes in its structure during its propagation. As the twin beam is endowed with multi-mode thermal statistics, the number *K* of modes can be estimated by the following formula that relies on the signal field:

25 $${K}^{n}=\frac{{({\sum }_{mlq}\langle {\hat{n}}_{s,mlq}\rangle )}^{2}}{{\sum }_{mlq}\,(\langle {\mathscr{N}}\,:{\hat{n}}_{s,mlq}^{2}\,:\rangle -{\langle {\hat{n}}_{s,mlq}\rangle }^{2})}=\frac{{({\sum }_{mlq}{V}_{mlq}^{2})}^{2}}{{\sum }_{mlq}\,{V}_{mlq}^{4}}.$$

## Mode Structure of Twin Beams

To reveal properties of pulsed intense twin beams generated in poled nonlinear crystals, we consider two crystals made of LiNbO_3_ and optimized for spectrally degenerate collinear down-conversion of type 0 (interaction among extraordinary waves) for the pump wavelength 775 nm and the signal and idler central wavelengths 1550 nm. The poling period Λ equals 9.516 *μ*m in this case. Both structures are composed of *N*_*L*_ = 700 domains and are approximately 6.66 mm long. Whereas the first regular periodical structure contains domains of the same length the second structure is chirped with chirping parameter *ζ* equal to 2.5 × 10^−6^ *μ*m^−1^ [*ζ*′ = 7.57 × 10^−6^ *μ*m^−2^]. In the chirped structure, the shortest (longest) domain length equals 9.036 *μ*m (9.995 *μ*m). Pump fields with radius *w*_p_ = 500 *μ*m, repetition rate *f* = 100 Hz, varying pump power *P* and three different spectral widths Δ*λ*_p_ equal to 0.2 nm (0.5 nm), 1 nm and 5 nm (FWHM, full width at half maximum, ${\rm{\Delta }}{\lambda }_{p}=4\pi c\sqrt{2\,\mathrm{ln}(2)}/[{({\omega }_{{\rm{p}}}^{0})}^{2}{\tau }_{{\rm{p}}}]$) are considered. The corresponding pump-pulse durations *τ*_p_ equal in turn 3.75 ps (1.5 ps), 0.75 ps and 0.15 ps. To distinguish the curves corresponding to different crystals and pump-pulse durations in the graphs below, we plot them in different colours: For the regular (chirped) structure, the curves determined for Δ*λ*_p_ = 0.2 nm (0.5 nm), 1 nm (1 nm) and 5 nm (5 nm) are in turn plotted in red (magenta), black (brown) and blue (green) colors. We note that both structures were analyzed in cw-regime in^15,28^ as sources of entangled photon pairs. In our analysis, we first address intensity parameters and mode structure of the emitted twin beams and then, in the following sections, we discuss their spectral and temporal properties as they vary with the increasing pump power *P*.

**Figure 1:**
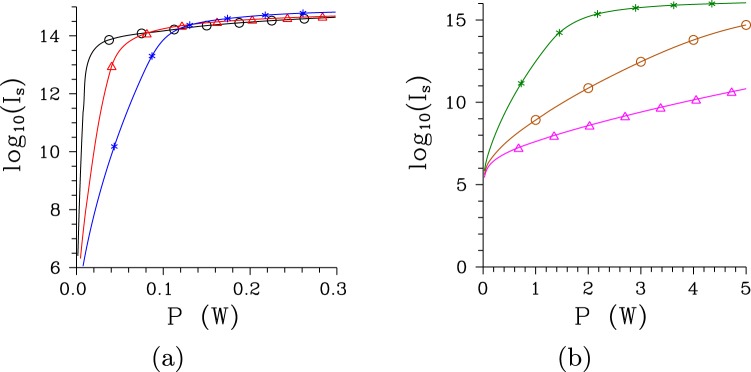
Signal intensities *I*_s_ as they depend on pump power *P* for (**a**) regular poled structure and Δ*λ*_p_ = 0.2 nm (red, $\bigtriangleup$), 1 nm (black, $\circ$) and 5 nm (blue, *) and (**b**) chirped poled structure and Δ*λ*_p_ = 0.5 nm (magenta, $\bigtriangleup$), 1 nm (brown, $\circ$) and 5 nm (green, *).

**Figure 2:**
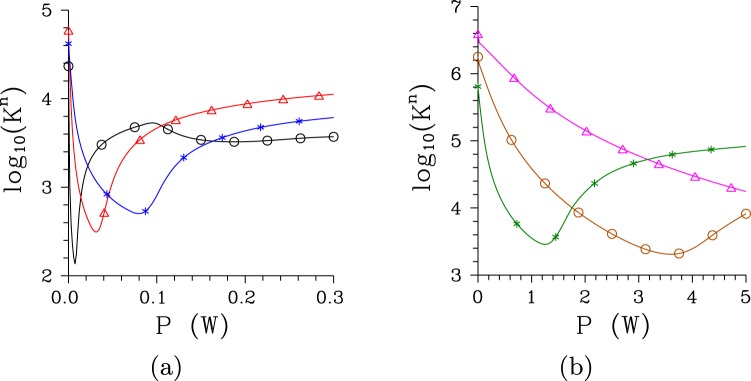
Numbers *K*^*n*^ of spatio-spectral modes as they depend on pump power *P* for (**a**) [(**b**)] regular [chirped] structure and Δ*λ*_p_ = 0.2 nm [0.5 nm] ($\bigtriangleup$), 1 nm ($\circ$) and 5 nm (*).

The number of signal photons (and thus also photon pairs) quantified by intensity *I*_s_ increases roughly exponentially with the increasing pump power *P* until certain threshold power *P*_th_ is reached. Then the increase of the number *I*_s_ of signal photons with the increasing power *P* is gradually becoming linear (see Fig. 1)^37^. The threshold power *P*_th_ considerably differs for both structures and it also depends on the pump spectral width Δ*λ*_*p*_. These dependencies can be understood by considering the dependence of the threshold pump power *P*_th_ on the number *K*^*n*^ of spatio-spectral modes that constitute the twin beam at low intensities. The greater the number *K*^*n*^ of such modes the greater the value of threshold power *P*_th_ (compare the curves in Figs 1 and 2). This behavior reflects the fact that the nonlinear evolution of a twin beam inside the crystal proceeds faster for the beams with lower numbers *K*^*n*^ of modes^34,37^. According to the theory presented in section Evolution of Intense Twin Beams, the pump, signal and idler fields can approximately be decomposed into many independent modes’ triplets each containing one pump, one signal and one idler spatio-spectral mode. Each modes’ triplet has its own effective nonlinear coupling constant and its pump mode contains certain fraction of the whole pump power *P*. For twin beams with lower numbers *K*^*n*^ of spatio-spectral modes greater pump powers are assigned to individual pump modes and this makes the nonlinear evolution along the crystal faster. The region with the exponential increase of intensity *I*_s_ naturally occurs for lower pump powers *P* at which individual signal and idler modes in each modes’ triplet exhibit an exponential increase. When the modes’ triplets with the greatest effective nonlinear coupling constants enter into the regime with pump mode depletion or even if the back-flow of energy from the signal and idler modes into the pump mode occurs, an exponential increase of intensity *I*_s_ is not more possible and the transition to the region with only a linear increase of intensity *I*_s_ of the whole signal field occurs.

As shown in Fig. 2 the number *K*^*n*^ of modes decreases with the increasing pump power *P* in the exponential region. This is a consequence of different effective nonlinear coupling constants assigned to different modes’ triplets and the nonlinear evolution that populates faster the signal and idler modes belonging to modes’ triplets with greater coupling constants^34,37^. This effect gradually diminishes the role of the signal and idler modes from modes’ triplets with smaller coupling constants in the twin beam and it leads to the dominance of the modes from the modes’ triplets with the greatest coupling constants. This dominance is maximal around the threshold power *P*_th_ as evidenced by the minimum of the number *K*^*n*^ of modes reached in this area. The loss of this dominance caused by pump-mode depletion and back-flow of energy in these modes’ triplets at the pump powers *P* greater than *P*_th_ allows the remaining modes to again significantly contribute to the twin-beam structure. This is reflected in the evolution of the number *K*^*n*^ of modes that increases with the increasing pump power *P* in this area with the typical linear increase of the signal intensity *I*_s_ (compare the curves in Figs 1 and 2).

**Figure 3:**
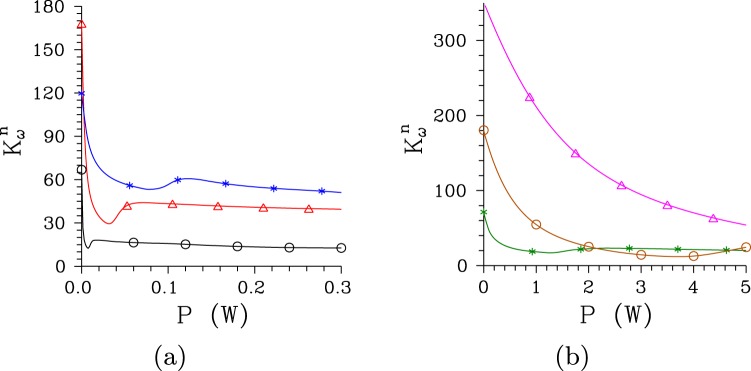
Numbers ${K}_{\omega }^{n}$ of spectral modes as they depend on pump power *P* for (a) [(b)] regular [chirped] structure and Δ*λ*_p_ = 0.2 nm [0.5 nm] ($\bigtriangleup$), 1 nm ($\circ$) and 5 nm (*).

The comparison of curves in Fig. 2(a,b) giving the numbers *K*^*n*^ of modes for the regular and chirped structure, respectively, reveals that, at low pump powers *P*, the twin beams in the chirped structure are composed of about two orders in magnitude greater numbers of spatio-spectral modes compared to those emitted from the regular structure. The nonlinear evolution dramatically decreases the number *K*^*n*^ of modes for both structures and the numbers *K*^*n*^ of modes are only by one order in magnitude greater for the chirped structure than those for the regular one for the pump powers *P* around *P*_th_. Whereas the twin beam generated by the pump field with Δ*λ*_p_ = 1 nm in the regular structure is effectively composed of 136 spatio-spectral modes at the threshold power *P*_th_ = 7.5 mW, 3380 modes are needed in case of the chirped structure for which the threshold power *P*_th_ = 3.9 W. In the regular (chirped) structure, as the pump power *P* changes from 0 to *P*_th_ the number ${K}_{\omega }^{n}$ of spectral modes is reduced from 66 (180) to 12 (15), the number ${K}_{k}^{n}$ of radial modes in the spatial spectrum lowers from 8 (37) to 1.7 (2.6) and the number ${K}_{\phi }^{n}$ of azimuthal modes in the spatial spectrum decreases from 40 (235) to 7 (85). The dependencies of the numbers ${K}_{\omega }^{n}$ of spectral modes on the pump power *P* with well defined minima are shown in Fig. 3 for both structures.

The number *K*^*n*^ of modes constituting a twin beam depends on the pump spectral width Δ*λ*_p_^35^. For a given structure there exists an optimal value of the pump spectral width Δ*λ*_p_ that gives the minimal number *K*^*n*^ of modes. So, both narrower and wider pump spectra lead to greater numbers *K*^*n*^ of modes, as documented in Figs 2(a) and 3(a) for the regular structure and Δ*λ*_p_ = 0.2 and 5 nm. Narrower pump spectra induce lower coherence in a twin beam whereas wider pump spectra lead to broader signal and idler spectra. In both cases a greater number *K*^*n*^ of modes is necessary to appropriately describe properties of the twin beam. As shown in Figs 2(b) and 3(b), broadening of the pump spectrum Δ*λ*_p_ from 1 nm to 5 nm improves coherence of the twin beam generated in the chirped structure such that a lower number *K*^*n*^ of modes is needed to built the twin beam.

## Spectral Properties of Twin Beams

**Figure 4:**
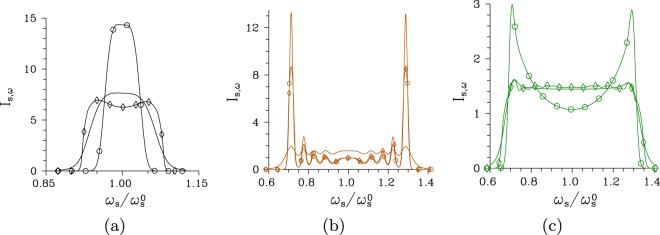
Temporal intensity spectra *I*_s,*ω*_ for (**a**) regular structure, Δ*λ*_p_ = 1 nm and *P* = 1 × 10^−5^ mW (plain curve), *P* = 7.5 mW ($\circ$) and *P* = 75 mW ($\diamond$) and (**b**) [(**c**)] chirped structure, Δ*λ*_p_ = 1 nm [Δ*λ*_p_ = 5 nm] and *P* = 1 × 10^−8^ W (plain curve), *P* = 2.9 W [*P* = 0.9 W] ($\circ$) and *P* = 5 W ($\diamond$). Intensity spectra *I*_s,*ω*_ are normalized such that $\int \,d{\omega }_{{\rm{s}}}{I}_{{\rm{s}},\omega }({\omega }_{{\rm{s}}})/{\omega }_{{\rm{s}}}^{0}=1$.

Changes in the mode structure of a twin beam considerably modify its spectrum and spectral coherence. Gradual increase of the pump power *P* makes the signal temporal intensity spectrum *I*_s,*ω*_ of a twin beam in the regular structure narrower until the threshold power *P*_th_ is reached. Then the signal intensity spectrum *I*_s,*ω*_ broadens and its central part is partially depleted due to the modes from modes’ triplets with the greatest coupling constants in which the back-flow of energy into the corresponding pump modes occurs [see Fig. 4(a)]. The spectral widths Δ*I*_s,*ω*_ depend on the pump spectral width Δ*λ*_p_: The wider the pump spectral width Δ*λ*_p_ the wider the signal spectral width Δ*I*_s,*ω*_. Whereas the low-intensity spectral widths Δ*I*_s,*ω*_ equal in turn 28, 33, and 68 nm considering Δ*λ*_p_ = 0.2, 1 and 5 nm and the regular structure, their narrowest widths observed around the threshold power *P*_th_ are 15.5, 18 and 36 nm. On the other hand, the temporal intensity spectra *I*_s,*ω*_ of twin beams in the chirped structure are broad (180–220 nm) for all considered pump spectral widths Δ*λ*_p_ = 0.5, 1 and 5 nm and these widths change only weakly as the pump power *P* increases [see Fig. 4(b,c)]. As typical for the chirped poled structures, these broad-band spectra contain local peaks that are considerably amplified as the pump power *P* increases [see Fig. 4(b)]. For the intensity spectra *I*_s,*ω*_ plotted in Fig. 4(b) for the pump spectral width Δ*λ*_p_ = 1 nm, the outer-most local peaks are magnified around six times at the threshold power *P*_th_. Wider pump spectra smooth out the local peaks in the signal low-intensity spectra *I*_s,*ω*_. Also the edges of intensity spectra *I*_s,*ω*_ are emphasized for the greater pump powers *P* around *P*_th_ in this case, as demonstrated in Fig. 4(c).

**Figure 5:**
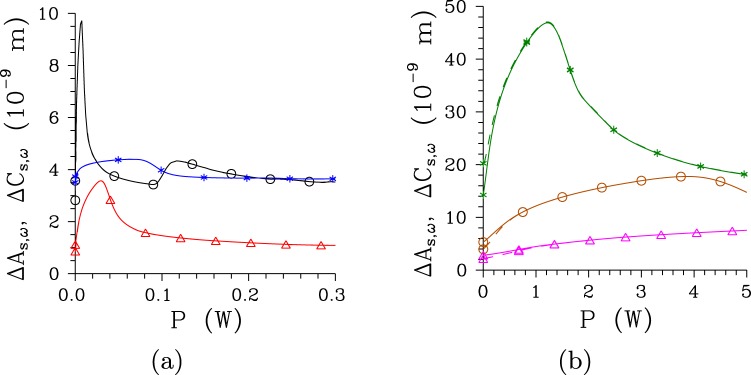
Widths Δ*A*_s,*ω*_ (solid curves) and Δ*C*_s,*ω*_ (dashed curves) of spectral intensity auto- and cross- correlation functions (FWHM) as they depend on pump power *P* for (**a**) [(**b**)] regular [chirped] structure and Δ*λ*_p_ = 0.2 nm [0.5 nm] ($\bigtriangleup$), 1 nm ($\circ$) and 5 nm (*). For greater *P*, solid and dashed curves nearly coincide.

Reduction of the number ${K}_{\omega }^{n}$ of spectral modes improves spectral coherence of a twin beam because the lowest-order spectral Schmidt modes dominate in the twin-beam structure and the suppressed higher-order modes do not degrade the spectral coherence. The widths Δ*A*_s,*ω*_ and Δ*C*_s,*ω*_ of spectral intensity auto- and cross-correlation functions, respectively, broaden from 3.5 [5.3] nm to 9.7 [17.8] nm as the pump power *P* increases towards *P*_th_ for the regular [chirped] structure and Δ*λ*_p_ = 1 nm (see Fig. 5). Moreover, considering the pump field with Δ*λ*_p_ = 5 nm and the chirped structure, the spectral widths Δ*A*_s,*ω*_ and Δ*C*_s,*ω*_ approach 47 nm for the threshold pump power *P*_th_, as it follows from the curves of Fig. 5(b).

**Figure 6:**
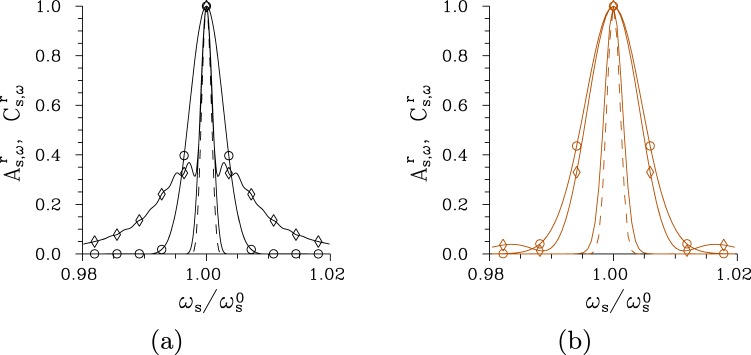
Relative spectral intensity auto- (${A}_{{\rm{s}},\omega }^{{\rm{r}}}$, solid curves) and cross- (${C}_{{\rm{s}},\omega }^{{\rm{r}}}$, dashed curves) correlation functions for (**a**) regular structure, Δ*λ*_p_ = 1 nm and *P* = 1 × 10^−5^ mW (plain curves), *P* = 7.5 mW ($\circ$) and *P* = 75 mW ($\diamond$) and (**b**) chirped structure, Δ*λ*_p_ = 1 nm and *P* = 1 × 10^−8^ W (plain curves), *P* = 2.9 W ($\circ$) and *P* = 5 W ($\diamond$); ${A}_{{\rm{s}},\omega }^{{\rm{r}}}({\omega }_{{\rm{s}}})\equiv {A}_{{\rm{s}},\omega }({\omega }_{{\rm{s}}},{\omega }_{{\rm{s}}}^{0})/{A}_{{\rm{s}},\omega }({\omega }_{{\rm{s}}}^{0},{\omega }_{{\rm{s}}}^{0})$, ${C}_{{\rm{s}},\omega }^{{\rm{r}}}({\omega }_{{\rm{s}}})\equiv {C}_{\omega }({\omega }_{{\rm{s}}},{\omega }_{{\rm{i}}}^{0})/{C}_{\omega }({\omega }_{{\rm{s}}}^{0},{\omega }_{{\rm{i}}}^{0})$. Solid and dashed curves with $\circ$ and $\diamond$ nearly coincide.

The spectral intensity auto-correlation functions *A*_s,*ω*_ are usually broader than their cross-correlation counterparts *C*_s,*ω*_ for low pump powers *P*, but they practically coincide for greater pump powers *P*, as shown in Fig. 5. Only for the chirped structure pumped by the field with a wide spectrum (Δ*λ*_p_ = 5 nm) the intensity cross-correlation function *C*_s,*ω*_ is broader than the corresponding auto-correlation function *A*_s,*ω*_ [see the curves with * in Fig. 5(b)]. This is a consequence of complex phase relations among the three interacting fields that originate both in the chirped structure and broad pump-field spectrum. Profiles of the spectral intensity auto- and cross-correlation functions *A*_s,*ω*_ and *C*_s,*ω*_ typical for low-intensity twin beams, twin beams generated around *P*_th_ and those observed for *P* > *P*_th_ are plotted in Fig. 6(a) for the regular structure and Fig. 6(b) for the chirped structure. We can see in these figures that broader tails are observed in the profiles for *P* > *P*_th_ and even well-developed side-peaks occur in the twin beams originating in the chirped structure.

It is remarkable that the curves in Fig. 5(a) giving the spectral widths Δ*A*_s,*ω*_ and Δ*C*_s,*ω*_ of the twin beam created in the regular structure by the pump field with Δ*λ*_p_ = 1 nm exhibit two coherence maxima, at the threshold powers *P*_th_ = 7.5 mW and 117 mW. The occurrence of multiple coherence maxima was discussed in^37^. These peaks are observed when the modes’ triplets with the greatest nonlinear coupling constants change the flow of energy more than one time during their propagation in the crystal. This leads to the presence of two considerably populated groups of modes in the twin-beam structure for the pump powers *P* around the second threshold which reduces the spectral coherence in this area compared to that found around the first threshold power.

**Figure 7:**
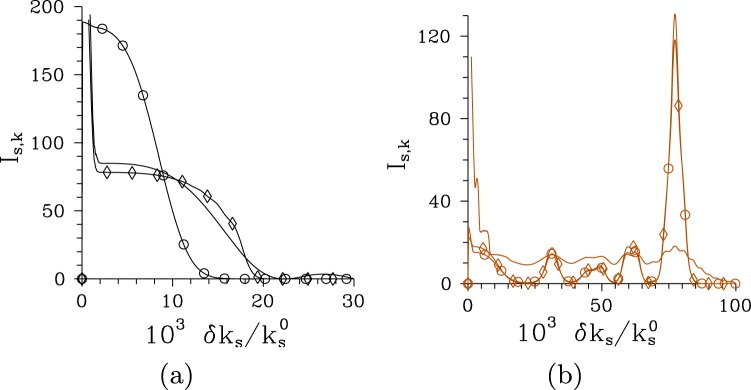
Radial spatial intensity spectra *I*_s,*k*_ for (**a**) regular structure and *P* = 1 × 10^−5^ mW (plain curve), *P* = 7.5 mW ($\circ$) and *P* = 75 mW ($\diamond$) and (**b**) chirped structure, *P* = 1 × 10^−8^ W (plain curve), *P* = 2.9 W ($\circ$) and *P* = 5 W ($\diamond$); Δ*λ*_p_ = 1 nm. Intensity spectra *I*_s,*k*_ are normalized such that $\int \,d{k}_{{\rm{s}}}^{\perp }{I}_{{\rm{s}},k}({k}_{{\rm{s}}}^{\perp })/{k}_{{\rm{s}}}^{0}=1$.

**Figure 8:**
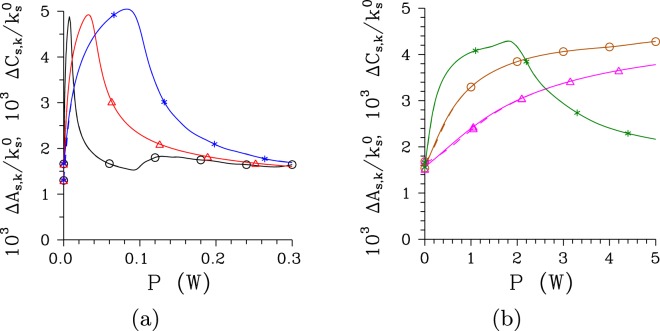
Widths Δ*A*_s,*k*_ (solid curves) and Δ*C*_s,*k*_ (dashed curves) of radial spatial intensity auto- and cross- correlation functions (FWHM) as they depend on pump power *P* for (**a**) [(**b**)] regular [chirped] structure and Δ*λ*_p_ = 0.2 nm [0.5 nm] ($\bigtriangleup$), 1 nm ($\circ$) and 5 nm (*). Solid and dashed curves nearly coincide.

The signal radial spatial intensity spectrum *I*_s,*k*_ determined in the transverse plane behaves qualitatively similarly as the temporal intensity spectrum *I*_s,*ω*_, as follows from the comparison of curves drawn in Figs 4 and 7 for the pump field with Δ*λ*_p_ = 1 nm and both analyzed structures. In the regular structure, the radial spatial intensity spectra *I*_s,*k*_ become narrower as the pump power *P* increases until *P*_th_ is reached and then they broaden. Contrary to this, the radial spatial intensity spectra *I*_s,*k*_ in the chirped structure keep their widths roughly the same as the pump power *P* grows. However, well separated peaks gradually develop as the pump power *P* increases [see Fig. 7(b)]. This means, that a low-intensity disc in the far-field plane of a twin beam is gradually transformed into concentric rings. Coherence of twin beams in the radial spatial spectrum as quantified by the intensity auto- and cross-correlation functions *A*_s,*k*_ and *C*_s,*k*_ considerably improves for pump powers around *P*_th_ compared to their low-intensity limits, as documented in Fig. 8. The comparison of curves drawn in Fig. 8 for different pump spectral widths Δ*λ*_p_ and both regular and chirped structures reveals that the maximal achievable spatial coherence of a twin beam is mainly determined by the pump beam radius *w*_p_.

## Temporal Properties of Twin Beams

**Figure 9:**
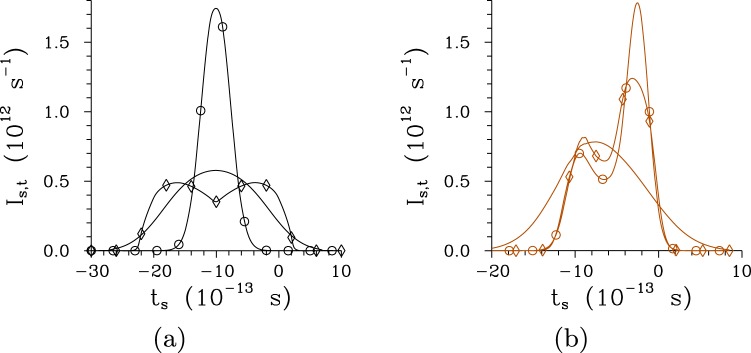
Signal photon fluxes *I*_s,*t*_ for (**a**) regular structure and *P* = 1 × 10^−5^ mW (plain curve), *P* = 7.5 mW ($\circ$) and *P* = 75 mW ($\diamond$) and (**b**) chirped structure and *P* = 1 × 10^−8^ W (plain curve), *P* = 2.9 W ($\circ$) and *P* = 5 W ($\diamond$); Δ*λ*_p_ = 1 nm. Photon fluxes *I*_s,*t*_ are normalized such that $\int \,d{t}_{{\rm{s}}}{I}_{{\rm{s}},t}({t}_{{\rm{s}}})=1$.

**Figure 10:**
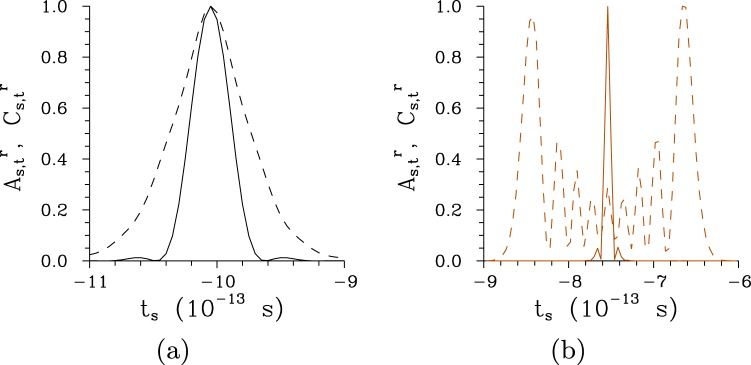
Relative temporal auto- [${A}_{{\rm{s}},t}^{{\rm{r}}}(t)\equiv {A}_{{\rm{s}},t}(t,{t}_{{\rm{s}}}^{{\rm{\max }}})/{A}_{{\rm{s}},t}({t}_{{\rm{s}}}^{{\rm{\max }}},{t}_{{\rm{s}}}^{{\rm{\max }}})$, solid curves] and cross- [${C}_{{\rm{s}},t}^{{\rm{r}}}(t)\equiv {C}_{t}(t,{t}_{{\rm{i}}}^{{\rm{\max }}})/{C}_{t}({t}_{{\rm{s}}}^{{\rm{\max }}},{t}_{{\rm{i}}}^{{\rm{\max }}})$, dashed curves) correlation functions of photon fluxes for (**a**) regular and (**b**) chirped structure; Δ*λ*_p_ = 1 nm, *P* = 1 × 10^−8^ W.

Photon fluxes of twin beams are considerably influenced both by the character of a poled structure as well as the pump power *P*. With the increasing pump power *P*, the profiles *I*_s,*t*_ of signal photon fluxes behave qualitatively in the same way as the corresponding temporal intensity spectra *I*_s,*ω*_ in the regular structure, as it follows from the comparison of curves in Figs 4(a) and 9(a). The profiles *I*_s,*t*_ of photon fluxes narrow as the pump power *P* increases for *P* < *P*_th_, then they broaden and later there occurs a dip in their central part due to pump depletion in individual modes’ triplets. In case of the chirped structure, the profiles *I*_s,*t*_ of photon fluxes slightly narrow as the pump power *P* increases and also local peaks are gradually formed in these profiles. Both structures generate the signal and idler pulsed fields around 1-ps long. The temporal auto-correlation functions *A*_s,*t*_ of photon fluxes are narrower than the corresponding temporal cross-correlation functions *C*_s,*t*_ both for the regular and chirped structure, as documented in Fig. 10. This relation is opposed to that observed between the spectral intensity auto- and cross-correlation functions. At low pump powers *P*, the widths Δ*A*_s,*t*_ of temporal auto-correlation functions belonging to the chirped structure are much narrower than the widths Δ*C*_s,*t*_ of the corresponding cross-correlation functions, and also considerably narrower than the widths Δ*A*_s,*t*_ determined for the twin beams in the regular structure. This indicates that the twin beams generated in the chirped structure, compared to those originating in the regular structure, will provide better temporal resolution in metrology applications including quantum optical coherence tomography^19^. Complex phase relations met during the twin-beam generation in the chirped structure may lead to oscillations in the temporal intensity correlation functions, as observed for the cross-correlation function *C*_s,*t*_ drawn in Fig. 10(b).

We note that the spectrally ultra broad-band photon pairs generated in chirped poled structures are conveniently used in the measurement of ultra-short temporal intervals^17^. It holds that the broader the spectrum, the shorter the smallest resolved temporal interval, that is reached after careful spectral phase compensation. From this point of view, the capability of more intense twin beams to resolve the shortest temporal intervals weakens for the pump powers around *P*_th_ as the nonlinear interaction makes the twin-beam spectra slightly narrower. On the other hand, the presence of a smaller number of spectral modes in the twin beams generated around the threshold power *P*_th_ partially reduces phase variations along the twin-beam spectra which results in a better temporal resolution achieved without applying an additional phase compensation.

**Figure 11:**
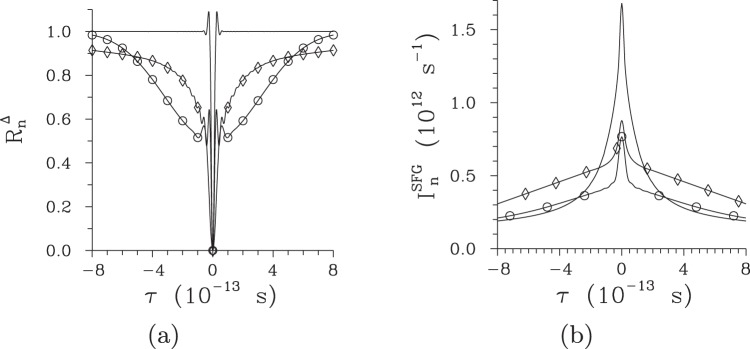
(**a**) Normalized interference pattern ${R}_{n}^{{\rm{\Delta }}}$ in the Hong-Ou-Mandel interferometer and (**b**) normalized sum-frequency intensity ${I}_{n}^{{\rm{SFG}}}$ as they depend on mutual delay *τ* between the signal and idler fields for regular structure and *P* = 1 × 10^−5^ mW (plain curve), *P* = 7.5 mW ($\circ$) and *P* = 75 mW ($\diamond$); Δ*λ*_p_ = 1 nm. It holds that $\int \,d\tau {I}_{n}^{{\rm{SFG}}}(\tau )=1$.

However, the analyzed temporal correlation functions cannot be observed directly. To reveal temporal coherence of a twin beam experimentally, one needs to rely either on the process of sum-frequency generation seeded by the twin beam or interference between the mutually delayed signal and idler fields in the Hong-Ou-Mandel interferometer^45^. In the Hong-Ou-Mandel interferometer whose interference pattern *R*^Δ^(*τ*) is described by Eq. (23), the analyzed twin beams reach 100% visibility due to their symmetry with respect to the signal and idler fields. Whereas the interference pattern *R*^Δ^ of low-intensity twin beams contains only a fast-oscillating narrow component in the central part, an additional broad component without oscillations occurs for more intense twin beams [see Fig. 11(a)]. The narrow component is typical for the Hong-Ou-Mandel interference observed with individual photon pairs^45^. On the other hand, the broad component is formed by the whole signal and idler fields and its contribution to the overall visibility is 50% for more intense twin beams. Similar components are identified also in the profile *I*^SFG^(*τ*) of sum-frequency intensity determined along Eq. (22) [see Fig. 11(b)]. Also here the narrow peak arises from the signal-idler fields’ correlations that originate in the pairing of photons. The broad peak arises from the usual sum-frequency response to two in general uncorrelated pulsed fields. While the broad dip in the Hong-Ou-Mandel interference pattern and broad peak in the sum-frequency intensity have comparable widths, the narrow dip in the interference pattern is narrower than the narrow peak of the sum-frequency intensity. For this reason, the use of Hong-Ou-Mandel interferometer in metrology for quantifying ultra-short temporal intervals gives better temporal resolution. The same conclusions can be drawn for the twin beams emitted from the chirped structure that provide even better temporal resolution. This is a consequence of considerably broader temporal spectra typical for the twin beams emitted from the chirped structures.

**Figure 12:**
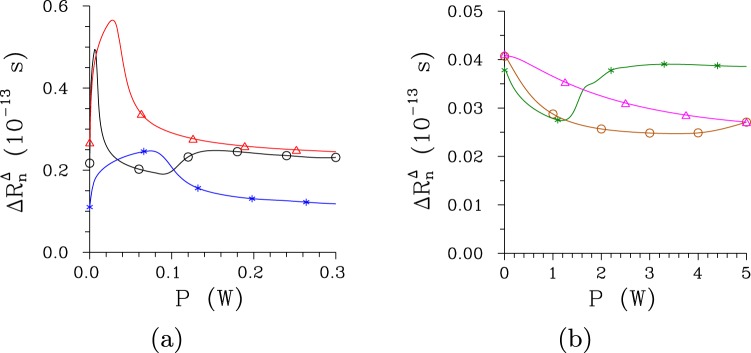
Widths ${\rm{\Delta }}{R}_{n}^{{\rm{\Delta }}}$ (FWHM) of the Hong-Ou-Mandel interference pattern as they depend on pump power *P* for (**a**) [(**b**)] regular [chirped] structure considering Δ*λ*_p_ = 0.2 nm [0.5 nm] ($\bigtriangleup$), 1 nm ($\circ$) and 5 nm (*).

As the pump power *P* increases, the widths of the broad dips (peaks) in the Hong-Ou-Mandel interference pattern (sum-frequency intensity) behave in the same way as the temporal widths of the signal photon fluxes. Similarly, the widths of the narrow dips (peaks) are close to the widths of temporal intensity auto-correlation functions. As a consequence, the narrow dips (peaks) become the widest for the pump powers *P* around *P*_th_, as illustrated in Fig. 12(a) for the regular structure and Hong-Ou-Mandel interference pattern. Whereas the narrow dip can be as short as 10 fs in the regular structure, the chirped structure allows for the generation of 3-fs-long dips [see Fig. 12(b)]. We note that the attainable accuracy in metrology applications based on such twin beams is a small fraction of these temporal intervals.

## Conclusions

The generation of intense twin beams in poled nonlinear crystals, both with periodic and chirped poling, has been analyzed. It has been shown that an initial exponential increase of twin-beam intensity with an increasing pump power is gradually suppressed and replaced by a linear increase. This allows to transfer a considerable amount of the pump energy into the twin beam. The increase of pump power gradually reduces the number of effectively populated spatio-spectral modes which is accompanied by the improvement of spatial and spectral coherence, both inside the signal and idler fields and between them. At certain threshold pump power, no further reduction of the number of twin-beam modes is possible and increase in the number of modes and degradation of twin-beam coherence follow for even greater pump powers. Nonlinear dynamics of modes in the twin beam and the pump field has been found to cause this behavior. Intense twin beams emitted from the chirped poled crystals have considerably broader spectra as well as larger spectral correlations compared to those generated in poled crystals with a periodic grating. This leads to considerably narrower temporal correlations that approach 3 fs in the analyzed cases. These ultra-short correlations are reached in the chirped poled crystals without considering any additional phase compensation, contrary to the case of the fields composed of individual photon pairs. These correlations may find their application in ultra-fast metrology based either on sum-frequency generation or the Hong-Ou-Mandel interferometer. The latter has been found more suitable owing to the better attainable temporal resolution.

## Data Availability

All data generated and analyzed during this study are included in this published article.
